# Supplementary material for: The Impact of Services on Economic Complexity: Service Sophistication as Route for Economic Growth
Source: PLoS One. 2016 Aug 25;11(8):e0161633. doi: 10.1371/journal.pone.0161633 (PMC4999235; doi:10.1371/journal.pone.0161633)
Supplement: S1 Supporting Information — Auxiliary results serving to assess the robustness of the results presented in the manuscript: Economic Complexity as Indicator of Future Growth: Robustness CheckAlternative RCA representation/Concatenated metrics (PDF) [file pone.0161633.s001.pdf]

S1 Supporting Information : The Impact of Services on Economic Complexity: Service Sophistication as Route for Economic Growth

Viktor Stojkoski[1], Zoran Utkovski[1,3], Ljupco Kocarev[1,2,*]

1 Research Center for Computer Science and Information Technologies, Macedonian Academy of Sciences and Arts, Skopje, Macedonia
2 Faculty of Computer Science and Engineering, Ss. Cyril and Methodius University in Skopje, Skopje, Macedonia
3 Faculty of Computer Science, University Goce Delčev in Štip, Štip, Macedonia

* E-mail: lkocarev@manu.edu.mk

1 Growth regressions: Robustness Check

We check the robustness of our econometric results by constructing a regression in which we exclude the period fixed effect, and by making three new regressions in which we introduce the initial export of goods and services (GS) as a percent of total GDP; the initial population; and the initial value added of services as a percent of total GDP (SVD) in the model. By excluding the period fixed effect we do not allow for changes in the average value of growth over time [1]. On the other hand, by including the aforementioned variables we account for possible effects of the trade openness, the size of the country and the magnitude of the service sector over growth. As stated in [2], for population, these variables may be interpreted as capabilities, and thus be correlated with the aggregated complexity measures. This implies that we might run into the problem of multicolinearity - failure to indicate significance of the correlated variables in the proposed model. Nonetheless, as presented in S1 Table 1, the aggregated complexity measures pass the test as significant predictors of long term economic even when discounting the period effect and adding the three other variables.

2 Alternative RCA representation/Concatenated metrics

Production of raw materials, manufactured goods and services are three separate processes of the economy (equivalent to the primary, secondary and tertiary sector of the economy). One may argue, due to often having vanishing fixed costs, that services have distinct export features, when compared to manufactured goods and raw materials whose cost of production can more easily be attributed to their corresponding export values. In this sense, export might no longer be an adequate presentation of the internal structure of the productive system of a country. In addition, as services may be a dominant part of some economic systems, their inclusion in the model might provide bias towards service-oriented economies. On the other hand, in spite of the potential drawbacks, it still may be argued that the concept of Revealed Comparative Advantage [3] is structured in a way that it still accounts for the varying export costs and export volumes among products (be it services and goods). With the aim to investigate these issues to more detail, here we consider an alternative representation where the RCA indices for services are calculated separately from the RCA indices for goods:

$$RCA_{is} = \frac{E_{is}/\sum_s E_{is}}{\sum_i E_{is}/\sum_{i,s} E_{is}};$$

$$RCA_{jg} = \frac{E_{jg}/\sum_g E_{jg}}{\sum_j E_{jg}/\sum_{j,g} E_{jg}},$$

where $s \in \{1, \ldots, S\}$ and $g \in \{1, \ldots, G\}$, with S and G denoting the number of services, respectively goods, in the model based on aggregated goods data. Afterwards, these indices are combined to construct the M matrix, from which "concatenated complexity measures" (i.e. ECI/linear PCI and Fitness/nonlinear PCI) are derived.

It can be argued that with this representation one *effectively* "divides" the economic system of a country into two sectors - services and goods. This may bias the results because when constructing the M matrix equal weights are given

S1 Table 1. Growth Regressions: Robustness check.

**Economic Complexity Index**					**Intensive Fitness**				
**Dependent Variable: Growth in GDP pc 1988-1998, 1998-2008**					**Dependent Variable: Growth in GDP pc 1988-1998, 1998-2008**				
**Variable**	(I)	(II)	(III)	(IV)	**Variable**	(I)	(II)	(III)	(IV)
Income per capita, logs	-0.044^{**}	-0.050^{***}	-0.037^{**}	-0.026	Income per capita, logs	-0.053^{***}	-0.053^{***}	-0.041^{***}	-0.032
	(0.014)	(0.016)	(0.015)	(0.023)		(0.014)	(0.016)	(0.015)	(0.020)
Increase in NR exports	0.453^{***}	0.322^{*}	0.329^{**}	0.302^{*}	Increase in NR exports	0.422^{***}	0.306^{*}	0.316^{*}	0.293^{***}
	(0.160)	(0.174)	(0.166)	(0.176)		(0.078)	(0.171)	(0.162)	(0.086)
aggregated ECI	0.046^{***}	0.045^{***}	0.036^{**}	0.035^{**}	aggregated IF, logs	0.060^{***}	0.048^{***}	0.040^{**}	0.049^{**}
	(0.014)	(0.015)	(0.015)	(0.018)		(0.017)	(0.017)	(0.019)	(0.021)
COI	0.005^{***}	0.004^{**}	0.005^{**}	0.004^{**}	COI	0.005^{**}	0.004^{**}	0.004^{**}	0.004^{**}
	(0.002)	(0.002)	(0.002)	(0.002)		(0.002)	(0.002)	(0.002)	(0.002)
Export of GS		0.001^{**}			Export of GS		0.001^{**}		
		(0.000)					(0.000)		
Population, logs			0.010		Population, logs			0.009	
			(0.014)					(0.015)	
SVD, logs				-0.092	SVD, logs				-0.102
				(0.129)					(0.093)
Constant	0.650^{***}	0.675^{***}	0.420	0.447^{*}	Constant	0.758^{***}	0.728^{***}	0.508	0.519^{**}
	(0.122)	(0.147)	(0.289)	(0.023)		(0.131)	(0.148)	(0.322)	(0.219)
Observations	210	201	210	178	Observations	210	201	210	178
R^2	0.178	0.240	0.243	0.242	R^2	0.196	0.242	0.244	0.252
Year FE	No	Yes	Yes	Yes	Year FE	No	Yes	Yes	Yes

For the regressions without fixed effects, ordinary standard errors are shown in parentheses. For all other regressions standard errors clustered by cross-section are shown. $^{***}p < 0.01$, $^{**}p < 0.05$, $^{*}p < 0.1$

to the overall exports of services and goods, thus potentially failing to acknowledge the initial differences in the nature and magnitude of their production. Moreover, it can be argued, that with this we slightly move away from the notion of Economic Complexity, since we implicitly assume that capabilities diffuse separately between goods and services (under the premise of a capability-driven interpretation of the country's productive structure).

In S1 Table 2 we reproduce the growth regressions with the concatenated complexity metrics. Column I states that the concatenated diversity is not a significant predictor of growth, whereas from columns II and IV we conclude that, on 5% level, the concatenated ECI and the concatenated IF significantly explain long term growth. When the models are compared, the the concatenated IF clearly outperforms the concatenated diversity, while the insignificance of the later in its individual model leads to insignificance of the model where we compare it to the concatenated ECI. As a means to infer which model performs better (whether the model with the concatenated ECI or the concatenated diversity) we must opt for another method for choosing between variables, such as the Information criterions. We estimated the Hannan-Quinn, Akaike and the Bayesian Information Criterion (not shown here, but available in the workfile and/or by request) and all of them prefer the model with the concatenated ECI.

Furthermore, in S1 Fig 1 we provide the yearly correlation between the aggregated complexity measures and the concatenated complexity measures based on aggregated data. Both, linear and nonlinear, aggregated and concatenated metrics have relatively high Spearman correlation over the years. Only after 2006 their correlation gradually declines and it ranges around 0.6 until the end of the period under investigation.

Finally, in S1 Fig 2 we show the yearly Spearman correlation between the disaggregated complexity measures and the concatenated complexity measures. These correlations are very similar to those presented in Fig 3 of the main manuscript, with only minor changes. In this figure in particular, in almost every year, and for both type of metrics (linear and nonlinear), the relations are relatively weaker. Only in the last two years (2009 and 2010), the correlation between the disaggregated and concatenated ECI does not fall as much as that for the disaggregated and aggregated ECI. On the other hand, the correlation between the disaggregated and concatenated IF is by far weaker than the one estimated for the disaggregated and aggregated IF.

The comparisons between the results presented in Fig 3 of the main manuscript and S1 Fig 2, can serve as a quantitative indicator for the conclusion that the aggregated measures, with the apparent drawback, are still a better approximation of the productive structure embedded in the goods and in the services and, as such, better indicators for the complexity of countries and products in the economic complexity terminology.

S1 Table 2. Concatenated Complexity Metrics and Growth

**Dependent Variable: Growth in GDP pc 1988-1998, 1998-2008**					
**Variable**	(I)	(II)	(III)	(IV)	(V)
Income per capita, logs	-0.032^{**}	-0.034^{***}	-0.035^{***}	-0.040^{***}	-0.042^{***}
	(0.014)	(0.014)	(0.014)	(0.015)	(0.015)
Increase in NR exports	0.303^{*}	0.318^{*}	0.318^{*}	0.302^{*}	0.300^{*}
	(0.165)	(0.167)	(0.167)	(0.164)	(0.167)
COI	0.003^{**}	0.004^{**}	0.004^{**}	0.004^{**}	0.004^{**}
	(0.002)	(0.002)	(0.002)	(0.002)	(0.002)
concatenated ECI		0.033^{**}	0.031		
		(0.016)	(0.019)		
concatenated IF, logs				0.034^{**}	0.070^{*}
				(0.016)	(0.040)
concatenated Diversity	0.008		0.001		-0.015
	(0.006)		(0.008)		(0.014)
Constant	0.508^{***}	0.562^{***}	0.562^{***}	0.626^{***}	0.729^{***}
	(0.118)	(0.126)	(0.127)	(0.138)	(0.172)
Observations	210	210	210	210	210
R^2	0.222	0.232	0.232	0.230	0.234
Year FE	Yes	Yes	Yes	Yes	Yes

Standard errors clustered by cross-section shown in parentheses. $^{***}p < 0.01$, $^{**}p < 0.05$, $^{*}p < 0.1$

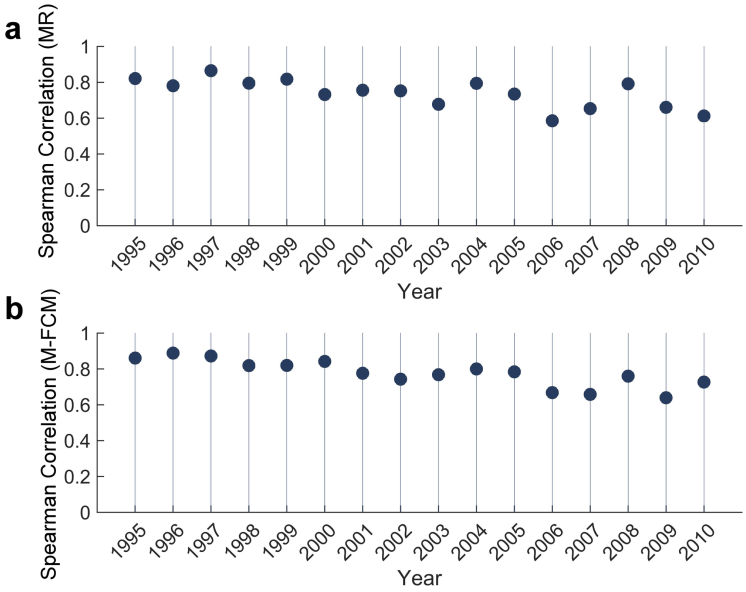

S1 Fig 1. Correlation between the aggregated and concatenated metrics. **a)** Yearly Spearman correlation between the aggregated and concatenated ECI (estimated through MR). **b)** same as **a)** for the aggregated and concatenated IF (estimated through M-FCM).

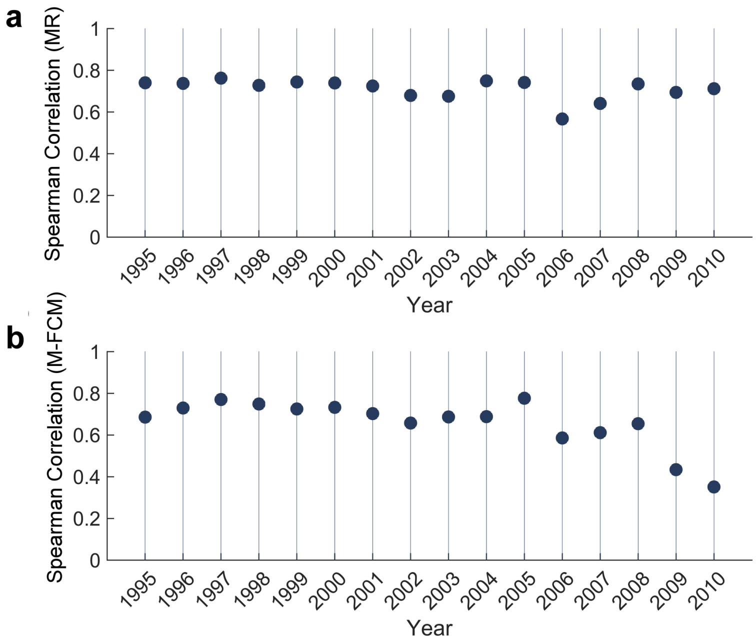

S1 Fig 2. Correlation between the disaggregated and concatenated metrics. **a)** Yearly Spearman correlation between the disaggregated and concatenated ECI (estimated through MR). **b)** same as **a)** for the disaggregated and concatenated IF (estimated through M-FCM).

References

1. Brooks C. Introductory econometrics for finance. Cambridge university press; 2014 May 2.

2. Cristelli M, Tacchella A, Pietronero L. The heterogeneous dynamics of economic complexity. PloS one. 2015 Feb 11;10(2):e0117174.

3. Balassa B. Trade liberalisation and "revealed" comparative advantage. The Manchester School. 1965 May 1;33(2):99-123.
